# Association between Dietary Fiber Intake and Physical Performance in Older Adults: A Nationwide Study in Taiwan

**DOI:** 10.1371/journal.pone.0080209

**Published:** 2013-11-11

**Authors:** I-Chien Wu, Hsing-Yi Chang, Chih-Cheng Hsu, Yen-Feng Chiu, Shu-Han Yu, Yi-Fen Tsai, Shi-Chen Shen, Ken N. Kuo, Ching-Yu Chen, Kiang Liu, Marion M. Lee, Chao A. Hsiung

**Affiliations:** 1 Institute of Population Health Sciences, National Health Research Institutes, Miaoli, County, Taiwan; 2 Program for Aging, College of Medicine, China Medical University, Taichung, Taiwan; 3 Chung Shan Medical University Hospital, Taichung, Taiwan; 4 Mennonite Christian Hospital, Hualien, Taiwan; 5 Center for Evidence-Based Medicine, Taipei Medical University, Taipei, Taiwan; 6 Department of Family Medicine, College of Medicine and Hospital, National Taiwan University, Taipei, Taiwan; 7 Department of Preventive Medicine, Northwestern University Feinberg School of Medicine, Chicago, Illinois, United States of America; 8 Department of Epidemiology and Biostatistics, University of California San Francisco, San Francisco, California, United States of America; Universidad Europea de Madrid, Spain

## Abstract

**Background:**

Physical performance is a major determinant of health in older adults, and is related to lifestyle factors. Dietary fiber has multiple health benefits. It remains unclear whether fiber intake is independently linked to superior physical performance. We aimed to assess the association between dietary fiber and physical performance in older adults.

**Methods:**

This was a cross-sectional study conducted with community-dwelling adults aged 55 years and older (n=2680) from the ongoing Healthy Aging Longitudinal Study (HALST) in Taiwan 2008-2010. Daily dietary fiber intake was assessed using a validated food frequency questionnaire. Physical performance was determined objectively by measuring gait speed, 6-minute walk distance, timed “up and go” (TUG), summary performance score, hand grip strength.

**Results:**

Adjusting for all potential confounders, participants with higher fiber intake had significantly faster gait speed, longer 6-minute walk distance, faster TUG, higher summary performance score, and higher hand grip strength (all *P* <.05). Comparing with the highest quartile of fiber intake, the lowest quartile of fiber intake was significantly associated with the lowest sex-specific quartile of gait speed (adjusted OR, 2.18 in men [95% CI, 1.33-3.55] and 3.65 in women [95% CI, 2.20-6.05]), 6-minute walk distance (OR, 2.40 in men [95% CI, 1.38-4.17] and 4.32 in women [95% CI, 2.37-7.89]), TUG (OR, 2.42 in men [95% CI, 1.43-4.12] and 3.27 in women [95% CI, 1.94-5.52]), summary performance score (OR, 2.12 in men [95% CI, 1.19-3.78] and 5.47 in women [95% CI, 3.20-9.35]), and hand grip strength (OR, 2.64 in men [95% CI, 1.61-4.32] and 4.43 in women [95% CI, 2.62-7.50]).

**Conclusions:**

Dietary fiber intake was independently associated with better physical performance.

## Introduction

Physical performance is a major determinant of health in older adults[1]. Good physical performance is critical if older adults wish to remain independent[2]. Moreover, physical performance at midlife and late life have recently been demonstrated to be novel and strong predictors of numerous major health outcomes in older adults[3]. The decline in objective measures of physical performance is believed to represent the early stage of a disablement process that leads to adverse health outcomes in older age[4]. For instance, a low summary performance score, short 6-minute walk distance, slow “timed up and go” (TUG), and low hand grip strength, are all independently associated with future disability, institutionalization and death[2,5-10]. One recent study showed that slow gait speed alone was an independent predictor of mortality in older people[11]. 

Detailed mechanisms underlying the prognostic significance of physical performance remain unclear. However, evidence suggests that poor physical performance is closely related to the aging process and subclinical conditions, and represents a state of inflammation and metabolic derangements[12-15]. An individual’s nutrition status is thought to play an important role in the pathogenesis of declining physical performance [4,16-19]; fortunately, nutrition is a modifiable lifestyle factor.

Dietary fiber comprises indigestible plant carbohydrates, and its intake is associated with multiple health effects[20,21]. Extensive research has demonstrated that fiber intake protects against common chronic diseases in older adults, including diabetes, obesity, and cardiovascular disease[21-23]. One recent study showed that dietary fiber can promote longevity[24]. Moreover, dietary fiber intake has been associated with a strong anti-inflammatory effect and beneficial metabolic effects, including a lowering of blood glucose and lipids[21]. Thus, it is likely that dietary fiber has a protective effect against physical performance impairment by influencing the pathways of aging, diseases processes, and metabolic or inflammatory activities.

This study investigated the relationship between dietary fiber intake and objective measures of physical performance in an Asian population. We hypothesized that low dietary fiber intake is independently associated with poor physical performance. To test this hypothesis, we analyzed the nutritional status in relation to their physical performance from a cross-sectional data of a nationwide observational study of community-dwelling older adults in Taiwan.

## Methods

### Ethics Statement

This study was approved by the institutional review boards (IRB) of National Health Research Institutes and Changhua Christian Hospital. The three local hospitals Yee Zen General Hospital, Hope Doctors Hospital and Potz Hospital recognized the IRB approval of National Health Research Institutes, since they do not have their own IRB. All participants signed informed consent forms at study entry.

### Participants

The Healthy Aging Longitudinal Study in Taiwan (HALST) is an ongoing population-based longitudinal study of adults aged 55 years and older. Data collection began in October, 2008. Being one of the few population-based long-term observational studies of aging in Taiwan, the study is designed to thoroughly examine the determinants of late-life health in an Asian population. A sample of community-dwelling older adults with diverse socio-demographic backgrounds was recruited from multiple areas across Taiwan, including 2 areas in the north region, 2 in central region, 2 in the south region, and one in the east region. 

In brief, a hospital was selected for each catchment area, and eligible residents living within about a 2-km radius of each hospital were ascertained. In order to recruit a sample consisting of elderly with different socio-demographic backgrounds, eligible residents in each geographic area were stratified according to age (55 to 64 y, and 65 y or older), sex and education levels. Subjects were then selected from each stratum by using the systemic random sampling method. To be eligible, a person was required to be 55 years or older and free of the following conditions: highly contagious infectious disease; severe illness (including malignancy undergoing active treatment); and severe hearing, speech, mental, or cognitive impairments. Adults who were bedbound or too frail to stand and ambulate, and adults who were institutionalized or hospitalized, were excluded from the study. At study entry, all participants received standardized physical performance assessments and laboratory examinations. They also completed interviewer-administered questionnaires to obtain information on sociodemographic status, health status, and lifestyle factors. During the laboratory examination, a blood sample was collected (after 8 hours or more of fasting) and was promptly centrifuged and stored at -80 °C. All blood sample analyses were performed in two central laboratories. Interviewers and laboratory personnel were all blinded to physical performance status of the participants.

We analyzed data from the baseline examinations conducted between October 2008 and October 2010, during which a cohort of 7060 adults was randomly sampled. A total of 6450 participants met our selection criteria, of whom 3121 agreed to participate. We further excluded 441 participants, yielding data for 2680 participants for analysis. Excluded were individuals too ill to undergo physical performance assessments (n= 216); and individuals who refused assessments (n= 225). Among these excluded participants, 38 individuals reported an unreliable or implausible dietary intake. 

### Assessment of Physical Performance

Physical performance was determined by measuring gait speed, 6-minute walk distance, TUG, summary performance score and hand grip strength. Gait speed was determined by observing participants walk 4 meters (m) at their usual pace, and timing the task according to a standardized protocol[2,11]. Time was measured by a trained examiner using a handheld stopwatch that measured to the nearest hundredth of a second (s). Participants were permitted to use a walking device such as a walker or cane. Gait speed was calculated as distance walked (m) divided by time (s).

The 6-minute walk test was performed according to a standardized protocol[6,25]. Briefly, participants were encouraged to walk as much distance as possible in 6 minutes. Although resting was allowed during the test, participants were instructed to resume walking as soon as possible. Total distance walked (m) was measured and recorded by a trained examiner.

For the TUG test, participants were observed standing up from a chair, walking 3 meters at their usual pace, turning around, and walking back to sit in the chair[26]. Time was measured by a trained examiner using a handheld stopwatch that recorded the nearest hundredth of a second.

Global lower extremity performance was assessed as a summary performance score using the Short Physical Performance Battery (SPPB)[2,27]. The SPPB includes the measurement of gait speed, standing balance, and time taken to rise 5 times from a chair. A score of 1 to 4 was assigned to each task with a higher score representing a better performance. The summary performance score was calculated by adding the scores for the 3 tasks. 

Hand grip strength was measured using a North Coast hand dynamometer (North Coast Medical Inc, Gilroy, CA, USA)[10]. Participants were seated in a chair with shoulder adducted and neutrally rotated and elbow flexed at 90°, and instructed to hold the dynamometer in the hand, and then to squeeze the dynamometer as hard as they could. Three trials were allowed for each hand in turn. The best performance, recorded in kg, was used for the analysis. 

### Assessment of Diet

Diet was assessed using a validated semiquantitative food frequency questionnaire, as described previously[28,29]. In brief, during face-to-face interviews, participants were asked of their usual intake frequency and portion size of 80 food and food items over the previous12 months. The responses for intake frequency were predefined and included “never or less than once per month”, “once per month”, “2-3 times per month”, “1-2 times per week”, “3-5 times per week”, “daily”, and “2 or more per day”. Appropriate food models were used to estimate the portion size of each food and food item. The intake of each nutrient per day (including fiber, calorie, protein, vitamin A, vitamin E, vitamin C, vitamin B6, and vitamin B12) was then computed from the estimated content of the relevant nutrient in specific foodstuffs, as reported by Taiwan’s Department of Health Nutrient Database of Taiwanese Foods[30]. An example of dietary fiber intake calculation is shown in Table S1. 

### Covariates

Covariates included the participant’s age; sex; marital status (unmarried, divorced, widowed, married); education level (

< high school, high school, > high school); smoking status (current, former, never); alcohol intake (drinker, non-drinker); physical activity (every day, less than every day, rarely or never); body mass index; status for metabolic syndrome; comorbidities; and the serum levels of high-sensitivity C-reactive protein (hs-CRP), a validated marker of chronic inflammation[31]. Participants who reported having smoked at least 100 cigarettes in their lifetime and were smokers at the time of the study were defined as current smokers, and participants who had smoked at least 100 cigarettes in their lifetime but no longer smoked cigarettes were classified as former smokers. Alcohol use classification was based on the National Institute on Alcohol Abuse and Alcoholism's guidelines for screening older adults for heavy drinking[32]. A non-drinker was defined as someone who reportedly had not consumed any alcoholic beverages in the past 12 months. Body mass index (BMI) was calculated as body weight (kg) divided by the square of the height (m^2^) and was categorized according to the WHO criteria recommended for Asians (overweight: 23 ≤ BMI < 27.5; obesity: BMI ≥ 27.5)[33]. Body weight and height were directly measured. A participant was considered to have metabolic syndrome if 3 or more of the following 5 criteria were present: elevated waist circumference (men ≥90cm; women ≥80cm); elevated triglycerides (≥150 mg/dl; or treatment for elevated triglycerides); reduced HDL cholesterol (men <40 mg/dl; women <50 mg/dl; or treatment for reduced HDL cholesterol); elevated blood pressure (systolic blood pressure ≥130 mm Hg; diastolic blood pressure ≥85 mm Hg; or treated hypertension); or elevated fasting glucose (glucose ≥100 mg/dl or treated diabetes)[34]. Blood glucose, triglyceride, and HDL cholesterol levels were determined enzymatically using the ADVIA^®^ 1800 Chemistry System (Siemens AG, Munich, Germany). The presence of hypertension was based on self-report, antihypertensive 

drug treatment, or an average blood pressure of 140 /90 mmHg or greater[35]. Diabetes mellitus was defined by self-report, medication use, fasting plasma glucose of 126 mg/dl or greater, or hemoglobin A1c of 6.5% or greater[36]. Hemoglobin A1c was measured by an ion exchange high-performance liquid chromatography using a HLC^®^-723G8 system (Tosoh Corporation, Tokyo, Japan). The Modification of Diet in Renal Disease (MDRD) equation proposed by Levey et al was used to estimate the glomerular filtration rate (GFR)[37]. Chronic kidney disease was defined as a GFR less than 60 ml·min^-1^ · (1.73 m^2^)^-1^[38]. Depressed mood was defined by a Center for Epidemiological Studies Depression Scale (CES-D) score of 20 or greater[39]. Other comorbidities were assessed by self-reported doctor's diagnosis, and included stroke, cardiovascular disease, arthritis, cancer, and lung disease. The serum high-sensitivity C-reactive protein (hs-CRP) was measured using latex-enhanced immunoturbidimetric assay using the ADVIA^®^ 1800 Chemistry System. The assay is capable of detecting CRP concentration as low as 0.012 mg/dl. The coefficient of variation was 3.78%.

### Statistical Analysis

Descriptive statistics were calculated to characterize the population. The results for all continuous variables are presented as mean ± SD. Median and interquartile range were used to present data that were skewed. Dietary factors were adjusted for total energy intake by the residual method[40]. Dietary fiber intake was further categorized into sex-specific quartiles. All analyses were stratified by sex. Differences in continuous variables among groups were analyzed with one-way analysis of variance (ANOVA), and differences in categorical variables (proportions) were analyzed with Chi-square tests. 

Linear regression analysis was performed to examine the associations between dietary fiber intake and physical performance and to estimate the adjusted physical performance. Tests for linear trend across quartiles of fiber intake were based on regression analyses, in which physical performance measures were regressed on the median values in each quartile of fiber intake. No major nonlinear associations were noted. Covariates were selected a priori based on documented associations with physical performance reported in the literature as well as biological plausibility. Covariates were organized into 5 related groups: age, risk factors for poor health (marital status, education level, smoking status, alcohol intake, physical activity, obesity, and metabolic syndrome), comorbidities (hypertension, diabetes mellitus, stroke, cardiovascular disease, arthritis, chronic kidney disease, cancer, lung disease, and depressed mood), nutrients (daily intake of energy, protein, vitamin A, vitamin E, vitamin C, vitamin B6, and vitamin B12), and inflammation (blood levels of hs-CRP). Using a forward stepwise method, adjustments were made for 5 models, with each successive model repeating the adjustments of the previous model, as follows: model *1* (adjustment for age), model *2* (plus adjustment for risk factors of poor health), model *3* (plus adjustment for comorbidities), model *4* (plus adjustment for nutrient intake), and model *5* (plus adjustment for inflammation). 

To further clarify how dietary fiber intake was related to physical performance, the associations were evaluated using forward stepwise multinomial logistic regression analysis with adjustment for covariates in model *4*. In this analysis, physical performance was coded into categories based on the quartile values within the sex-specific study cohort. For all analyses, differences were considered significant if *P* < .05. We calculated 95% confidence intervals (CI) and reported the CI for each parameter estimate. All the analyses were performed using SPSS version 16.0 (SPSS Inc., Chicago, IL, USA). 

## Results

Table 1 displays the characteristics of the participants by sex. For age, the mean ± SD was 69 ± 8 years; and 53% of participants were women. For fiber intake (energy-adjusted), the mean ± SD was 29 ± 13 g/d, ranging from 13 g/d (10^th^ percentile) to 47 g/d (90^th^ percentile) in men, and from 17 to 45 g/d in women. Table 2 shows the characteristics of men and women stratified by quartiles of dietary fiber intake. Participants with a low fiber intake tended to be older, less educated, and physically inactive, and were more likely to have lower intakes of protein, vitamin A, vitamin E, vitamin C, and vitamin B6; they were also more likely to exhibit a higher level of hs-CRP. 

**Table 1:** Population Characteristics of the Study Participants^*^.

		**Men**	**Women**
		**n= 1271**	**n= 1409**
Age, mean (SD), y		69 (8)	69 (8)
Marital status ^†^			
	Unmarried, n (%)	16 (1.3)	18 (1.3)
	Divorced, n (%)	20 (1.6)	23 (1.6)
	Widowed, n (%)	116 (9.1)	450 (31.9)
Education ^†^			
	< High school, n (%)	637 (50.1)	1027 (72.9)
	High school, n (%)	418 (32.9)	277 (19.7)
	> High school, n (%)	216 (17.0)	105 (7.5)
Smoking ^†^			
	Never, n (%)	524 (41.2)	1390 (98.7)
	Previous smoker, n (%)	408 (32.1)	6 (0.4)
	Current smoker, n (%)	339 (26.7)	13 (0.9)
Alcohol use, n (%) none ^†^		736 (57.9)	1202 (85.3)
Exercise			
	Every day, n (%)	691 (54.4)	784 (55.6)
	Less than every day, n (%)	203 (16.0)	247 (17.5)
	Rarely or never, n (%)	377 (29.7)	378 (26.8)
Metabolic syndrome, n (%) ^†^		511 (40.2)	761 (54)
Obesity, n (%)		226 (17.8)	264 (18.7)
Hypertension, n (%)		648 (51)	726 (51.5)
Diabetes mellitus, n (%)		367 (28.9)	386 (27.4)
Stroke, n (%) ^†^		74 (5.8)	39 (2.8)
Cardiovascular disease, n (%)		232 (18.3)	273 (19.4)
Arthritis, n (%) ^†^		143 (11.3)	338 (24)
Chronic kidney disease, n (%) ^†^		187 (14.7)	126 (8.9)
Cancer, n (%) ^†^		49 (3.9)	81 (5.7)
Lung disease, n (%) ^†^		62 (4.9)	34 (2.4)
Depressed mood, n (%) ^†^		29 (2.3)	62 (4.4)
hs-CRP, mg/dL ^‡^		0.06 (0.02-0.21)	0.07 (0.02-0.21)
Daily dietary intake ^§^			
	Fiber, g/d ^†^	28.40 (13.97)	29.82 (11.78)
	Total energy, kcal/d ^†^	2175.8 (733.2)	1727.3 (609.4)
	Protein, g/d	82.1 (21.3)	82.2 (17.0)
	Vitamin A, RE/d ^†^	4684.74 (4299)	6225.28 (4759.88)
	Vitamin E, α-TE/d ^†^	4.11 (2.04)	3.57 (1.36)
	Vitamin C, mg/d ^†^	220.05 (137.84)	270.49 (137.46)
	Vitamin B6, mg/d ^†^	1.95 (0.55)	1.88 (0.41)
	Vitamin B12, μg/d ^†^	6.00 (6.02)	5.45 (3.71)
Physical performance			
	Gait speed, m/s ^†^	0.89 (0.23)	0.79 (0.23)
	Six-minute walk distance, m ^†^	394 (81)	361 (79)
	Timed up and go, s ^†^	11.9 (3.7)	13.0 (4.5)
	Summary performance score ^†^	9.4 (2.1)	8.4 (2.2)
	Hand grip strength, kg ^†^	37.3 (8.4)	22.7 (5.6)

* Data are means (standard deviation) unless otherwise specified (N= 2680).

^†^ Significant difference between men and women (*P* <.05). Continuous variables were analyzed with *t* tests, while categorical variables (proportions) were analyzed with Chi-square test.

^‡^ Median (interquartile range).

^§^ Except for total energy intake, all nutrients were adjusted for total energy intake[40]. To convert kilocalories to kilojoules, multiply by 4.186. RE indicates retinol equivalents; and α-TE, α-tocopherol equivalents.

**Table 2:** Characteristics of Study Participants by Dietary Fiber Intake^*^.

		**Dietary Fiber Intake, Quartiles** ^†^				
**Characteristics**		**Q1**	**Q2**	**Q3**	**Q4**	***P*** ^‡^
**Men**						
Age, y		69.2 (8.9)	70.2 (8.2)	69.8 (7.7)	67.9 (7.3)	.002
Marital status						.38
	Unmarried, n (%)	3 (0.9)	7 (2.2)	3 (0.9)	3 (0.9)	
	Divorced, n (%)	9 (2.8)	4 (1.3)	3 (0.9)	4 (1.3)	
	Widowed, n (%)	32 (10.1)	32 (10.1)	29 (9.1)	23 (7.3)	
Education						.04
	< High school, n (%)	173 (54.4)	172 (54.1)	157 (49.4)	135 (42.6)	
	High school, n (%)	96 (30.2)	102 (32.1)	105 (33.0)	115 (36.3)	
	> High school, n (%)	49 (15.4)	44 (13.8)	56 (17.6)	67 (21.1)	
Smoking						.84
	Never, n (%)	131 (41.2)	133 (41.8)	139 (43.7)	121 (38.2)	
	Previous smoker, n (%)	99 (31.1)	99 (31.1)	99 (31.1)	111 (35.0)	
	Current smoker, n (%)	88 (27.7)	86 (27.0)	80 (25.2)	85 (26.8)	
Alcohol use, n (%) none		195 (61.3)	190 (59.7)	181 (56.9)	170 (53.6)	.22
Exercise						<.001
	Every day, n (%)	141 (44.3)	167 (52.5)	188 (59.1)	195 (61.5)	
	Less than every day, n (%)	59 (18.6)	43 (13.5)	59 (18.6)	42 (13.2)	
	Rarely or never, n (%)	118 (37.1)	108 (34.0)	71 (22.3)	80 (25.2)	
Metabolic syndrome, n (%)		108 (34.0)	136 (42.8)	124 (39.0)	143 (45.1)	.03
Obesity, n (%) ^§^		46 (14.5)	53 (16.7)	56 (17.6)	71 (22.4)	.02
Hypertension, n (%)		137 (43.1)	171 (53.8)	163 (51.3)	177 (55.8)	.008
Diabetes, n (%)		79 (24.8)	92 (28.9)	100 (31.4)	96 (30.3)	.28
Stroke, n (%)		23 (7.2)	18 (5.7)	15 (4.7)	18 (5.7)	.60
Heart disease, n (%)		50 (15.7)	69 (21.7)	57 (17.9)	56 (17.7)	.26
Arthritis, n (%)		29 (9.1)	43 (13.5)	38 (11.9)	33 (10.4)	.33
CKD, n (%)		46 (14.5)	65 (20.4)	44 (13.8)	32 (10.1)	.003
Cancer, n (%)		12 (3.8)	12 (3.8)	14 (4.4)	11 (3.5)	.94
Lung disease, n (%)		15 (4.7)	14 (4.4)	18 (5.7)	15 (4.7)	.90
Depressed mood, n (%)		17 (5.3)	6 (1.9)	3 (0.9)	3 (0.9)	<.001
hs-CRP, mg/dL ^║^		0.07 (0.02-0.19)	0.08 (0.03-0.24)	0.06 (0.02-0.22)	0.05 (0.02-0.22)	.04^¶^
Daily dietary intake						
	Energy, kcal/d	2385.3 (752.3)	2017.2 (705.1)	2046.7 (723.4)	2254.3 (690.3)	<.001
	Protein, g/d	76.1 (26.1)	79.2 (16.8)	83.2 (18.6)	90.0 (20.1)	<.001
	Vitamin A, RE/d	1909.85 (2746.50)	4049.46 (2911.28)	5244.67 (3848.15)	7543.98 (5165.32)	<.001
	Vitamin E, α-TE/d	3.00 (1.35)	3.41 (1.27)	4.23 (1.53)	5.82 (2.48)	<.001
	Vitamin C, mg/d	104.48 (74.66)	186.74 (71.19)	241.42 (92.95)	347.96 (159.97)	<.001
	Vitamin B6, mg/d	1.67 (0.61)	1.82 (0.45)	1.99 (0.42)	2.31 (0.49)	<.001
	Vitamin B12, μg/d	6.63 (7.25)	5.65 (3.91)	6.08 (4.86)	5.65 (7.29)	.13
Physical performance						
	Gait speed, m/s	0.84 (0.23)	0.86 (0.23)	0.92 (0.23)	0.93 (0.22)	<.001
	Six-minute walk distance, m	380 (85)	382 (82)	403 (78)	409 (77)	<.001
	Timed up and go, s	12.5 (4.5)	12.4 (4.3)	11.6 (3.2)	11.1 (2.5)	<.001
	Summary performance score	9.1 (2.2)	9.1 (2.2)	9.7 (1.9)	9.7 (2.0)	<.001
	Hand grip strength, kg	35.6 (8.6)	36.2 (8.4)	37.5 (7.7)	39.9 (8.2)	<.001
**Women**						
Age, y		69.9 (8.0)	69.8 (7.8)	69.0 (7.8)	67.2 (7.3)	<.001
Marital status						.43
	Unmarried, n (%)	7 (2.0)	3 (0.8)	3 (0.9)	5 (1.4)	
	Divorced, n (%)	6 (1.7)	5 (1.4)	6 (1.7)	6 (1.7)	
	Widowed, n (%)	124 (35.2)	100 (28.3)	122 (34.7)	104 (29.5)	
Education						<.001
	< High school, n (%)	288 (81.8)	265 (75.1)	253 (71.9)	221 (62.8)	
	High school, n (%)	41 (11.6)	68 (19.3)	75 (21.3)	93 (26.4)	
	> High school, n (%)	23 (6.5)	20 (5.7)	24 (6.8)	38 (10.8)	
Smoking						.33
	Never, n (%)	347 (98.6)	348 (98.6)	346 (98.3)	349 (99.1)	
	Previous smoker, n (%)	1 (0.3)	1 (0.3)	4 (1.1)	0 (0)	
	Current smoker, n (%)	4 (1.1)	4 (1.1)	2 (0.6)	3 (0.9)	
Alcohol use, n (%) none		310 (88.1)	315 (89.2)	306 (86.9)	271 (77.0)	<.001
Exercise						
	Every day, n (%)	160 (45.5)	190 (53.8)	217 (61.6)	217 (61.6)	<.001
	Less than every day, n (%)	55 (15.6)	66 (18.7)	52 (14.8)	74 (21.0)	
	Rarely or never, n (%)	137 (38.9)	97 (27.5)	83 (23.6)	61 (17.3)	
Metabolic syndrome, n (%)		190 (54.0)	188 (53.3)	200 (56.8)	183 (52.0)	.62
Obesity, n (%) ^§^		59 (16.8)	59 (16.7)	77 (21.9)	69 (19.6)	.13
Hypertension, n (%)		195 (55.4)	180 (51.0)	177 (50.3)	174 (49.4)	.39
Diabetes, n (%)		90 (25.6)	105 (29.7)	95 (27.0)	96 (27.3)	.66
Stroke, n (%)		12 (3.4)	11 (3.1)	12 (3.4)	4 (1.1)	.19
Heart disease, n (%)		76 (21.6)	58 (16.4)	76 (21.6)	63 (17.9)	.20
Arthritis, n (%)		84 (23.9)	78 (22.1)	93 (26.4)	83 (23.6)	.60
CKD, n (%)		33 (9.4)	32 (9.1)	36 (10.2)	25 (7.1)	.52
Cancer, n (%)		17 (4.8)	19 (5.4)	26 (7.4)	19 (5.4)	.48
Lung disease, n (%)		11 (3.1)	7 (2.0)	7 (2.0)	9 (2.6)	.72
Depressed mood, n (%)		28 (8.0)	11 (3.1)	15 (4.3)	8 (2.3)	.001
hs-CRP, mg/dL ^║^		0.08 (0.02-0.25)	0.07 (0.02-0.23)	0.07 (0.03-0.22)	0.06 (0.02-0.18)	.03^¶^
Daily dietary intake						
	Energy, kcal/d	1816.6 (583.9)	1619.0 (535.8)	1594.8 (583.8)	1879.0 (677.8)	<.001
	Protein, g/d	74.0 (16.9)	80.0 (13.7)	84.6 (15.0)	90.2 (17.8)	<.001
	Vitamin A, RE/d	2968.78 (1916.13)	5270.89 (2613.94)	6683.36 (2880.25)	9980.78 (6794.05)	<.001
	Vitamin E, α-TE/d	2.94 (0.81)	3.26 (0.81)	3.63 (1.08)	4.46 (1.92)	<.001
	Vitamin C, mg/d	145.66 (60.75)	221.59 (56.68)	289.11 (62.23)	425.75 (149.85)	<.001
	Vitamin B6, mg/d	1.66 (0.39)	1.80 (0.35)	1.91 (0.33)	2.15 (0.42)	<.001
	Vitamin B12, μg/d	5.29 (4.58)	5.40 (2.59)	5.67 (3.78)	5.43 (3.61)	.57
Physical performance						
	Gait speed, m/s	0.73 (0.21)	0.77 (0.23)	0.79 (0.23)	0.86 (0.22)	<.001
	Six-minute walk distance, m	332 (81)	354 (79)	367 (77)	391 (66)	<.001
	Timed up and go, s	14.1 (6.3)	13.2 (4.1)	13.0 (4.0)	11.7 (2.7)	<.001
	Summary performance score	7.6 (2.3)	8.2 (2.2)	8.5 (2.1)	9.2 (1.9)	<.001
	Hand grip strength, kg	21.3 (5.5)	22.3 (5.7)	23.0 (5.8)	24.2 (5.0)	<.001

* Data are means (SD) unless otherwise specified. CKD indicates chronic kidney disease; Hs-CRP, high sensitivity C-reactive protein; RE, retinol equivalents; and α-TE, α-tocopherol equivalents.

^†^ In men, Q1 = <19.18 g/day (n= 318), Q2= 19.18-26.38 g/day (n=318), Q3= 26.39-35.12 g/day (n=318), Q4= >35.12 g/day (n=317). In women, Q1 = <21.42 g/day (n=352), Q2= 21.42-28.06 g/day (n=353), Q3= 28.07-35.35 g/day (n=352), Q4= >35.35 g/day (n=352).

^‡^ Continuous variables were analyzed with One-way Analysis of Variance, while categorical variables (proportions) were analyzed with Chi-square test.

^§^ World Health Organization’s recommended criteria for Asians (23 ≤ BMI < 27.5 for overweight and BMI ≥ 27.5 for obesity)[33].

^║^ Median (interquartile range).

^¶^ Test for trend.

We found that participants with a lower fiber intake had significantly slower gait speed, shorter six-minute walk distance, slower TUG, lower summary performance score, and weaker hand grip strength (Table 2). Linear regression analyses revealed a strong relationship between dietary fiber intake and all physical performance measures, for both men and women (all *P* < .001). For both men and women, dietary fiber intake remained significantly associated with gait speed, 6-minute walk distance, TUG, summary performance score, and hand grip strength after adjustments for the following variables: age (model *1*;all *P* < .005), risk factors for poor health (model *2*; all *P* < .01), and comorbidities (model *3*; all *P* < .05). Additional adjustment for nutrient intake did not change the associations between fiber intake and physical performance (model *4*; all *P* <.05). Participants with higher fiber intake had significantly faster gait speed, longer 6-min walk distance, faster TUG, higher summary performance score, and higher hand grip strength (Table 3). Compared with men in the highest quartile for dietary fiber intake (>35.12 g/d), men in the lowest quartile for fiber intake (<19.18 g/d) had a mean 0.09 m/s slower gait speed (*P* < .001); a mean 23.8 m shorter 6-min walk distance (*P* < .001); a mean 1.2 s longer TUG (*P* < .001); a mean 0.6 lower summary performance score (*P* < .001); and a mean 3.7 kg weaker hand grip strength (*P* < .001). Similarly, compared with women in the highest quartile for dietary fiber intake (>35.35 g/d), women in the lowest quartile for fiber intake (<21.42 g/d) had a mean 0.08 m/s slower gait speed (*P* < .001); a mean 32.8 m shorter 6-min walk distance (*P* < .001); a mean 1.5 s longer TUG (*P* < .001); a mean 1.0 lower summary performance score (*P* < .001); and a mean 1.9 kg weaker hand grip strength (*P* < .001).

**Table 3:** Adjusted Relationships between Amount of Dietary Fiber Intake and Physical Performance^*^.

	**Quartile of Dietary Fiber Intake** ^†^				
**Physical Performance**	**Q1**	**Q2**	**Q3**	**Q4**	***P* for trend**
**Men**					
Gait speed, m/s	0.80 (0.02)	0.83 (0.02)	0.89 (0.02)	0.89 (0.02)	<.001
Six-minute walk distance, m	356.6 (7.3)	367.4 (7.3)	381.9 (7.3)	380.4 (7.3)	<.001
Timed up and go, s	13.6 (0.3)	13.1 (0.3)	12.5 (0.3)	12.4 (0.3)	<.001
Summary performance score	7.7 (0.2)	7.9 (0.2)	8.1 (0.2)	8.3 (0.2)	.001
Hand grip strength, kg	31.2 (1.1)	32.7 (1.1)	33.8 (1.1)	34.9 (1.1)	<.001
**Women**					
Gait speed, m/s	0.72 (0.02)	0.76 (0.02)	0.77 (0.02)	0.80 (0.03)	<.001
Six-minute walk distance, m	322.3 (8.3)	339.7 (8.5)	349.0 (8.3)	355.1 (8.5)	<.001
Timed up and go, s	14.4 (0.4)	13.5 (0.4)	13.4 (0.4)	12.9 (0.4)	<.001
Summary performance score	7.5 (0.2)	8.0 (0.2)	8.2 (0.2)	8.5 (0.2)	<.001
Hand grip strength, kg	19.4 (0.5)	20.4 (0.5)	20.8 (0.5)	21.3 (0.5)	<.001

* Data are means (SE), and are adjusted for age, marital status, education level, smoking status, alcohol intake, physical activity, body mass index, metabolic syndrome, hypertension, diabetes mellitus, stroke, cardiovascular disease, arthritis, chronic kidney disease, cancer, lung disease, depressed mood, dietary intake of energy, protein, vitamin A, vitamin E, vitamin C, vitamin B6 and vitamin B12.

^†^ In men, Q1 = <19.18 g/day, Q2= 19.18-26.38 g/day, Q3= 26.39-35.12 g/day, Q4= >35.12 g/day. In women, Q1 = <21.42 g/day, Q2= 21.42-28.06 g/day, Q3= 28.07-35.35 g/day, Q4= >35.35 g/day.

Multinomial logistic regression analysis confirmed the independent association between dietary fiber intake and physical performance, after adjustment for all covariates, for both men (Table 4) and in women (Table 5). For men, those with a fiber intake less than 19.18 g/d had at least a 2-fold increase for the following odds: gait speed less than 0.75 m/s; 6-minute walk distance less than 349 m; TUG longer than 13.3 s; summary performance score less than 9; and hand grip strength less than 33 kg, compared with men whose dietary fiber intake was more than 35.12 g/d (Table 4). For women, those with a fiber intake less than 21.42 g/d had at least a 3-fold increase for the following odds: gait speed less than 0.63 m/s; 6-minute walk distance less than 315 m; TUG longer than 14.6 s; summary performance score less than 8; and hand grip strength less than 20 kg, compared with women whose dietary fiber intake was more than 35.35 g/d (Table 5). Among women, different fiber intake was not associated with different prevalence of metabolic syndrome, obesity, hypertension and chronic kidney disease. Analysis was repeated in women without adjustment for metabolic syndrome, BMI, hypertension and chronic kidney disease. Since there were no changes in risk estimates, we present results after adjustment for all covariates.

**Table 4:** Odds Ratio of Poorer Physical Performance by Quartile of Dietary Fiber Intake, Relative to the Best Performance in Men^*^.

		**Quartile of Dietary Fiber Intake (g/d)**				
**Physical Performance**		**Q1 (<19.18)**	**Q2 (19.18-26.38)**	**Q3 (26.39-35.12)**	**Q4 (>35.12)**	***P***
Gait speed, m/s						.002
	<0.75 relative to >1.04	2.18 (1.33-3.55)	1.36 (0.85-2.17)	0.82 (0.51-1.31)	1.00 (Reference)	
	0.75-0.88 relative to >1.04	1.88 (1.19-2.98)	1.21 (0.78-1.89)	0.76 (0.49-1.18)	1.00 (Reference)	
	0.89-1.04 relative to >1.04	1.63 (1.01-2.61)	0.95 (0.60-1.50)	1.07 (0.70-1.64)	1.00 (Reference)	
Six-minute walk distance, m						.001
	<349 relative to >448	2.40 (1.38-4.17)	2.20 (1.29-3.76)	1.04 (0.61-1.78)	1.00 (Reference)	
	349-394 relative to >448	1.43 (0.85-2.39)	1.34 (0.81-2.21)	1.25 (0.78-1.99)	1.00 (Reference)	
	395-448 relative to >448	1.11 (0.69-1.79)	0.84 (0.52-1.35)	0.76 (0.49-1.18)	1.00 (Reference)	
Timed up and go, s						.02
	>13.3 relative to <9.7	2.42 (1.43-4.12)	1.70 (1.00-2.88)	1.33 (0.79-2.25)	1.00 (Reference)	
	11.3-13.3 relative to <9.7	1.26 (0.79-2.01)	1.14 (0.72-1.80)	0.81 (0.51-1.28)	1.00 (Reference)	
	9.7-11.2 relative to <9.7	0.98 (0.62-1.54)	0.99 (0.64-1.55)	1.04 (0.68-1.59)	1.00 (Reference)	
Summary performance score						.009
	<9 relative to >11	2.12 (1.19-3.78)	1.27 (0.74-2.17)	0.86 (0.50-1.48)	1.00 (Reference)	
	9-10 relative to >11	1.67 (0.99-2.82)	0.90 (0.55-1.47)	0.91 (0.56-1.48)	1.00 (Reference)	
	11 relative to >11	1.30 (0.74-2.29)	0.64 (0.37-1.11)	1.00 (0.60-1.67)	1.00 (Reference)	
Hand grip strength, kg						.005
	<33 relative to >42	2.64 (1.61-4.32)	1.89 (1.16-3.10)	1.27 (0.78-2.08)	1.00 (Reference)	
	33-37 relative to >42	1.78 (1.10-2.88)	1.71 (1.04-2.80)	1.58 (0.99-2.52)	1.00 (Reference)	
	38-42 relative to >42	1.28 (0.81-2.02)	1.30 (0.83-2.03)	1.20 (0.78-1.85)	1.00 (Reference)	

* Data are odds ratios (95% confidence interval) of poorer physical performance (lower three quartiles of gait speed, six-minute walk distance, summary performance score and hand grip strength and higher three quartiles of timed up and go) with respect to the best performance (highest quartile of gait speed, six-minute walk distance, summary performance score and hand grip strength and lowest quartile of timed up and go), and are adjusted for age, marital status, education level, smoking status, alcohol intake, physical activity, body mass index, metabolic syndrome, hypertension, diabetes mellitus, stroke, cardiovascular disease, arthritis, chronic kidney disease, cancer, lung disease, depressed mood, dietary intake of energy, protein, vitamin A, vitamin E, vitamin C, vitamin B6 and vitamin B12.

**Table 5:** Odds Ratio of Poorer Physical Performance by Quartile of Dietary Fiber Intake, Relative to the Best Performance in Women^*^.

	**Quartile of Dietary Fiber Intake (g/d)**				
**Physical Performance**	**Q1 (<21.42)**	**Q2 (21.42-28.06)**	**Q3 (28.07-35.35)**	**Q4 (>35.35)**	***P***
Gait speed, m/s					<.001
<0.63 relative to >0.93	3.65 (2.20-6.05)	1.96 (1.20-3.20)	1.91 (1.16-3.12)	1.00 (Reference)	
0.63-0.78 relative to >0.93	2.07 (1.32-3.24)	1.08 (0.70-1.68)	1.13 (0.73-1.74)	1.00 (Reference)	
0.79-0.93 relative to >0.93	1.49 (0.96-2.34)	1.34 (0.89-2.02)	1.30 (0.87-1.96)	1.00 (Reference)	
Six-minute walk distance, m					.001
<315 relative to >414	4.32 (2.37-7.89)	2.22 (1.25-3.92)	1.65 (0.96-2.86)	1.00 (Reference)	
315-367 relative to >414	2.05 (1.22-3.45)	1.41 (0.87-2.28)	1.05 (0.66-1.66)	1.00 (Reference)	
368-414 relative to >414	1.51 (0.92-2.48)	1.45 (0.93-2.25)	1.22 (0.80-1.86)	1.00 (Reference)	
Timed up and go, s					.003
>14.6 relative to <10.3	3.27 (1.94-5.52)	2.20 (1.30-3.71)	1.96 (1.16-3.31)	1.00 (Reference)	
12.1-14.6 relative to <10.3	1.56 (0.98-2.47)	1.12 (0.71-1.78)	1.15 (0.73-1.80)	1.00 (Reference)	
10.3-12.0 relative to <10.3	1.22 (0.79-1.89)	1.29 (0.85-1.95)	1.20 (0.80-1.81)	1.00 (Reference)	
Summary performance score					<.001
<8 relative to >10	5.47 (3.20-9.35)	2.62 (1.60-4.28)	2.07 (1.28-3.36)	1.00 (Reference)	
8 relative to >10	2.72 (1.53-4.84)	1.49 (0.86-2.57)	1.33 (0.78-2.26)	1.00 (Reference)	
9-10 relative to >10	2.01 (1.24-3.27)	1.42 (0.92-2.20)	1.32 (0.87-2.01)	1.00 (Reference)	
Hand grip strength, kg					<.001
<20 relative to >26	4.43 (2.62-7.50)	1.62 (0.99-2.66)	1.47 (0.88-2.44)	1.00 (Reference)	
20-22 relative to >26	1.93 (1.20-3.10)	0.79 (0.51-1.23)	0.83 (0.53-1.31)	1.00 (Reference)	
23-26 relative to >26	1.75 (1.11-2.76)	0.72 (0.47-1.11)	1.13 (0.75-1.71)	1.00 (Reference)	

* Data are odds ratios (95% confidence interval) of poorer physical performance (lower three quartiles of gait speed, six-minute walk distance, summary performance score and hand grip strength and higher three quartiles of timed up and go) with respect to the best performance (highest quartile of gait speed, six-minute walk distance, summary performance score and hand grip strength and lowest quartile of timed up and go), and are adjusted for age, marital status, education level, smoking status, alcohol intake, physical activity, body mass index, metabolic syndrome, hypertension, diabetes mellitus, stroke, cardiovascular disease, arthritis, chronic kidney disease, cancer, lung disease, depressed mood, dietary intake of energy, protein, vitamin A, vitamin E, vitamin C, vitamin B6 and vitamin B12.

To determine whether any relationships existed between dietary fiber intake and physical performance independently of inflammation, we performed linear regression analysis with adjustment for inflammation marker levels (*model 5*). For both men and women, fiber intake continued to show significant associations to gait speed (all *P* <.05), six-minute walk distance (all *P* <.05), timed up and go (all *P* <.05), summary performance score (all *P* <.05) and hand grip strength (all *P* <.05).

## Discussion

In this large observational study of older adults in Taiwan, we found that low dietary fiber intake was associated with poor physical performance, regardless of which objective measures were used to assess physical performance. The associations were independent of any known risk factors for poor physical performance, including sociodemographic factors, lifestyle-related factors, comorbidities, and intake of other nutrients.

Our study demonstrated that lower dietary fiber intake was associated with slower gait speed, shorter 6-minute walk distance, slower timed “up and go”, lower summary performance score, and weaker hand grip strength. These findings were congruent with the results of other recent studies[41,42]. Tomey et al prospectively examined the relationship between dietary intake at midlife and reported disability 4 years later[42]; low fiber intake at baseline was found to be associated with future functional limitations. An ealier prospective study had shown that low intake of fruit and vegetable at midlife was a risk factor for disability[41]. These studies had indicated that dietary fiber plays an important but unknown role in the development of disability in the second half of a person’s lifetime. Our findings suggested that inadequte dietary fiber intake might contribute to the disablement process through an impairment of physical performance. Individuals with low fiber intake (less than 19 g/day in men and less than 21g/day in women) were more likely to have gait speed less than 0.6 m/sec, timed up and go more than 15 sec, summary performance score less than 8 and hand grip strength less than 33 kg in men and 20 kg in women. Adults with these poor physical performances are known to be at high risk of disability[2,7,10,43-47]. In addition, the differences in physical performance measures between participants in the highest and lowest fiber intake categories were consistent with clinically meaningful differences, further highlighting the clinical significance of our results[48-50]. 

The independent nature of the relationship between dietary fiber intake and physical performance is biologically understandable. The amount of fiber present in a person’s diet is strongly and inversely related to the degree of systemic inflammation. Human intervention trials have shown that solely by increasing the daily fiber intake, systemic inflammation can be reduced[51]. The current study confirmed that participants with lower fiber intake had higher levels of serum hs-CRP. Lower fiber intake may thus lead to a greater degree of systemic inflammation, which in turn contributes to poorer physical performance[14,15]. In addition, dietary fiber exerts numerous beneficial metabolic effects[21]. Improved glucose and lipid metabolism, together with a reduction in hyperglycemia and hypercholesterolemia, as well as improved insulin sensitivity, have been observed in people following a high fiber intake[52,53]. Thus, inadequate fiber intake exacerbates poor glucose and lipid metabolism and results in a decline of physical performance[15,54]. However, we observed that the association between low fiber intake and poor physical performance remained significant after adjustment for inflammation and metabolic markers. This finding indicates that additional dietary fiber-related pathways play a role in maintaining physical performance. Dietary fiber exerts a major effect on the microbes living in the human gastrointestinal tract (gut microbiota) [55-57], which increasing evidence has identified as an important determinant of human health[57,58].

The strengths of the current study were as follows. To gain an in-depth understanding of the relationship between diet and physical performance, we examined more than one objective measure of physical performance. To clarify the independent role of fiber intake in physical performance, we carefully controlled for conditions that might affect physical performance. We further controlled for physiological variables, including inflammation and metabolic markers, to investigate how dietary fiber is related to physical performance. Finally, the relationship between nutritional status and physical function has been examined mainly among Western populations. This might be the first study to explore the relationship between dietary fiber and physical performance using a large representative sample of an Asian population; dietary habits differ between Asians and Westerners. 

The study was subject to certain limitations. Its cross-sectional design meant that we were unable to establish temporal relationships and infer causal links between dietary fiber intake and physical performance. It is reasonable to believe the lifelong dietary habit precedes performance changes at old age, but it is also possible that older adults with poor physical performance have difficulties in maintaining a healthy diet. In addition, confounding variables might have affected the outcome measures. Although we attempted to include all possible confounders in the analysis, residual confounding by unmeasured variables (e.g., detailed health-related behaviors, other micronutrients and dietary factors) is likely. In particular, low intake of dietary antioxidants is a known risk factor for decline in physical function[18,19], and may confound the observed link between dietary fiber intake and physical performance. In assessing the participant’s disease status, we relied mainly on self-reports of the presence of disease. Subclinical conditions might thus have been missed. Also of concern was potential information bias, which might occur if frail older participants recalled their food consumption, disease history, or health-related lifestyle status less accurately than healthier participants. The HALST cohort consists mainly of community-dwelling older adults, who might be healthier than the age-matched general population. Thus, the findings of this study might not be generalizable to the entire older population. Lastly, the levels of dietary intake in our study should be interpreted with caution. HALST participants might have over-reported their food intakes on the semiquantitative food frequency questionnaire[59]. Although a semiquantitative food frequency questionnaire may yield different estimates of the actual daily foods intake as compared with other methods, it has reasonable levels of reproducibility and validity, and performs well in ranking individuals by intake levels, making it useful in large scale epidemiologic studies examining the relationships between diet and disease risk[28,29,60-62]. 

Further prospective observational studies are needed to confirm the relationships we observed. Intervention trials might be warranted to determine whether increasing people’s fiber intake reduces their functional decline. In addition, more detailed research is required to address the mechanisms linking dietary fiber intake to physical performance. These efforts will contribute to the development of novel preventive and therapeutic strategies for frail older adults.

In conclusion, dietary fiber intake is independently and positively associated with physical performance in older adults. Dietary fiber may offer protection against disability associated with old age. 

## Supporting Information

Table S1 **An Example of Dietary Fiber Intake Calculation.**
(DOC)
