# Supplementary material for: Association between Dietary Fiber Intake and Physical Performance in Older Adults: A Nationwide Study in Taiwan
Source: PLoS One. 2013 Nov 11;8(11):e80209. doi: 10.1371/journal.pone.0080209 (PMC3823869; doi:10.1371/journal.pone.0080209)
Supplement: Table S1 — An Example of Dietary Fiber Intake Calculation. (DOC) [file pone.0080209.s001.doc]

**Table S1. An Example of Dietary Fiber Intake Calculation**

| **Food Items** | **Fiber Consumption Via Food Item (g) / day** |
| --- | --- |
| Banana | 0.16 |
| Apple | 1.06 |
| Pear | 1.53 |
| Papaya | 1.17 |
| Pineapple | 0.14 |
| Mango | 0.01 |
| Kiwi fruit | 0.12 |
| Guava | 6.00 |
| Watermelon/melon | 0.13 |
| Orange/grapefruit | 1.04 |
| Grape | 0.14 |
| Dark colored vegetables | 13.68 |
| Bean sprout | 0.02 |
| Light-colored vegetables | 2.06 |
| Carrot | 3.12 |
| Radish | 0.89 |
| Tomato | 0.12 |
| Eggplant | 3.15 |
| Asparagus/bamboo shoot/water bamboo | 0.57 |
| Gourd (e.g. wax gourd, sponge gourd, cucumber) | 0.78 |
| Onion | 0.41 |
| Pumpkin/sweet potato | 0.55 |
| Ginger/garlic | 0.30 |
| Beans (e.g. vegetable soybean, broad bean, pea, snap bean.) | 1.11 |
| Sea weed/laver | 1.60 |
| Pickled vegetables | 0.15 |
| Eggs (scrambled, fried or steamed egg) | 0.07 |
| Soybean products | 0.93 |
| Oil-fried soybean products | 0.01 |
| Red bean/green bean | 0.00 |
| Miso/tofu soup | 0.01 |
| Soybean milk/tofu pudding | 0.00 |
| Nuts (peanut, walnut) | 2.10 |
| Cooked rice | 0.01 |
| Germ rice/brown rice | 2.81 |
| Oatmeal | 0.18 |
| Noodle/noodle string | 0.15 |
| Mung bean thread | 0.02 |
| Potato | 0.01 |
| Corn | 0.03 |
| Toast/bread | 0.29 |
| Steamed bread | 0.28 |
| Bun/dumpling/spring roll | 0.46 |
| Sesame pancake/scallion pancake | 0.01 |
| Instant noodles | 0.03 |
| Chinese style pastries | 0.01 |
| Western style pastries | 0.11 |
| Tong sui | 0.01 |
| OoLong tea/other tea | 0.00 |
| Vegetable and fruit juice | 0.03 |
| Total | 47.56 |
